# Two Novel Mutations in the *JAG1* Gene in Pediatric Patients with Alagille Syndrome: The First Case Series in Czech Republic

**DOI:** 10.3390/diagnostics11060983

**Published:** 2021-05-28

**Authors:** Dagmar Procházková, Romana Borská, Lenka Fajkusová, Petra Konečná, Eliška Hloušková, Zdeněk Pavlovský, Ondřej Slabý, Šárka Pospíšilová

**Affiliations:** 1 Department of Pediatrics, Faculty of Medicine, University Hospital Brno, Masaryk University, 625 00 Brno, Czech Republic; konecna.petra3@fnbrno.cz (P.K.); Hlouskova.Eliska@fnbrno.cz (E.H.); 2 Department of Clinical Genetics and Genomics, Faculty of Medicine, University Hospital Brno, Masaryk University, 625 00 Brno, Czech Republic; pospisilova.sarka@fnbrno.cz; 3 Center of Molecular Biology and Genetics, Department of Internal Medicine, Hematology and Oncology, Faculty of Medicine, University Hospital Brno, Masaryk University, 625 00 Brno, Czech Republic; borska.romana@fnbrno.cz (R.B.); fajkusova.lenka@fnbrno.cz (L.F.); 4 Department of Pathology, Faculty of Medicine, University Hospital Brno, Masaryk University, 625 00 Brno, Czech Republic; pavlovsky.zdenek@fnbrno.cz; 5 Department of Biology, Faculty of Medicine, Masaryk University, 625 00 Brno, Czech Republic; oslaby@med.muni.cz; 6 Central European Institute of Technology, Masaryk University, 625 00 Brno, Czech Republic

**Keywords:** Alagille syndrome, *JAG1* gene, pediatric patients, cholestasis

## Abstract

Background: Alagille syndrome (ALGS) is a highly variable multisystem disorder inherited in an autosomal dominant pattern with incomplete penetration. The disorder is caused by mutations in the *JAG1* gene, only rarely in the *NOTCH2* gene, which gives rise to malformations in multiple organs. Bile duct paucity is the main characteristic feature of the disease. Methods: Molecular-genetic examination of genes *JAG1* and *NOTCH2* in four probands of Czech origin who complied with the diagnostic criteria of ALGS was performed using targeted next-generation sequencing of genes *JAG1* and *NOTCH2.* Segregation of variants in a family was assessed by Sanger sequencing of parental DNA. Results: Mutations in the *JAG1* gene were confirmed in all four probands. We identified two novel mutations: c.3189dupG and c.1913delG. Only in one case, the identified *JAG1* mutation was de novo. None of the parents carrying *JAG1* pathogenic mutation was diagnosed with ALGS. Conclusion: Diagnosis of the ALGS is complicated due to the absence of clear genotype-phenotype correlations and the extreme phenotypic variability in the patients even within the same family. This fact is of particular importance in connection to genetic counselling and prenatal genetic testing.

## 1. Introduction

To find the cause of infantile cholestatic jaundice is often difficult in clinical practice, especially after ruling out common problems. It may be found in disorders of bile acid synthesis or secretion, hereditary fructose intolerance, mitochondrial hepatopathy, progressive familial intrahepatic cholestasis (including Byler), as well as in conditions characterised by biliary hypoplasia, e.g., Alagille syndrome (ALGS).

Alagille syndrome is a rare, highly variable, autosomal dominant multisystem disorder caused by defects in the Notch signalling pathway. We distinguish ALGS type 1 (ALGS1) (MIM#118450), which is due to mutation in the *JAG1* gene on chromosome 20p12, the prevalence of which is 1:30,000 of live births [1,2,3,4]. The other is ALGS type 2, (ALGS2) (MIM#610205), which is associated with mutation in the *NOTCH2* gene on chromosome 1p12 and is a rarer form of the disorder (1:70,000 live-born children). About 1% of the probands have mutations in the *NOTCH2* gene [5]. The only study to directly estimate the incidence of ALGS was published in 1977 by Danks et al. [6]. However, it was conducted before a molecular genetic diagnosis was possible. ALGS is likely underdiagnosed in the absence of molecular genetic conformations [7].

Up to now, mutations in gene *JAG1* have been discovered in 70–94% of ALGS patients. The vast majority of *JAG1* mutations are truncating mutations (nonsense or frameshift) or whole/partial gene deletions, which can be located across the extracellular domain of the protein [7].

**Figure 1:**
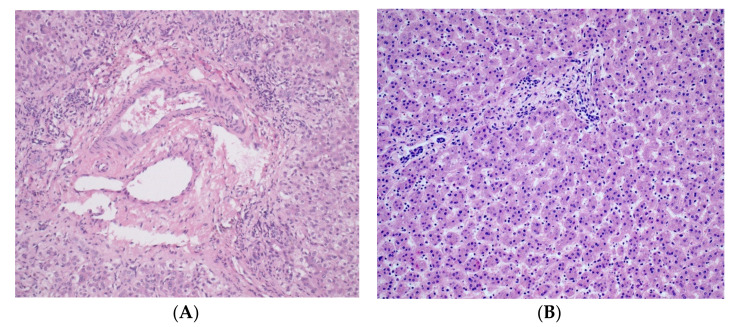
(**A**) Histopathology of liver showing portobiliary area without intrahepatic bile ducts (H&E staining at 200× magnification, patient no. 4); (**B**) Healthy liver tissue: hepatic parenchyma formed by regular single-row beams of bland hepatocytes and unexpanded portobiliary areas (H&E staining at 200× magnification).

**Figure 2:**
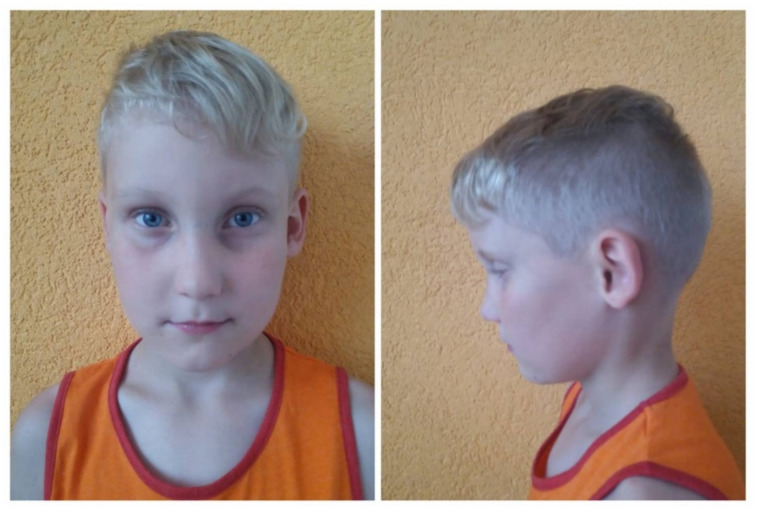
The ALGS1 patient: characteristic triangle-shaped facial appearance with a broad forehead, deeply-set eyes, hypertelorism, low-set ears and long onion-shaped nose (patient no. 2).

The basic symptom of ALGS is the reduced number of bile ducts (bile duct paucity) (Figure 1A) within the liver combined with five diagnostic signs: cholestasis, congenital heart defects (most frequently peripheral pulmonary stenosis), abnormalities of the skeleton (most often butterfly vertebrae), eye defects (usually embryotoxon posterior), and characteristic triangle-shaped facial appearance with a broad forehead, deeply-set eyes, hypertelorism, low-set ears, and long onion-shaped nose (Figure 2). The diagnosis is confirmed if three of the five signs are present.

Clinically, ALGS is a heterogeneous defect. Several larger descriptive studies consistently showed a significant degree of renal and vascular involvement. About 39% of the patients suffer from renal damage, most frequently renal dysplasia [8,9,10,11,12]. Some patients show vascular anomalies, often in the head and neck regions [13,14]. Infrequently, we observe retarded psycho-motor development and learning disability [15]. Pancreatic insufficiency or growth retardation was observed in individual cases. 

More recent studies suggest that renal and vascular abnormalities should be considered as part of the disease-defining criteria, and that the diagnosis can be made when three out of seven exist [7].

In the present study, we described the phenotype of four child probands with ALGS1; molecular-genetic examinations confirmed their affliction.

## 2. Material and Methods

The study was conducted with four patients of Czech origin examined at the Department of Pediatrics of the University Hospital in Brno, who complied with the diagnostic criteria of ALGS, and with 8 of their relatives. Before we started the study, we obtained the approval of the children’s parents and of adults who participated in the study. The study was conducted in accord with ethical principles of the Helsinki Declaration and was approved by the ethical committee of the University Hospital in Brno.

### Mutation Analysis

For identification of sequence variants associated with ALGS, we used the solution-based capture method SeqCap EZ Choice Library (Roche Nimble-Gene, Madison, WI, USA) and targeted resequencing on the NextSeq (Illumina, San Diego, CA, USA). A custom capture array was designed to capture exons and adjacent intron sequences of 187 genes of which 2 genes are associated with ALGS (*JAG1* and *NOTCH2*). The technique was described in more detail in our previous studies [16,17,18]. Identified sequence changes were filtered against an in-house list of common gene variants and the frequencies of candidate disease variants were also determined in the ExAC Browser (http://exac.broadinstitute.org), Exome Variant Server (http://evs.gs.washington.edu/EVS/), and the 1000 GenomesProject (www.1000genomes.org/). Identified pathogenic and probably pathogenic sequence variants were confirmed by PCR and Sanger sequencing using the BigDye Terminator Cycle Sequencing Kit (Applied Biosystems, Waltham, MA, USA) on the ABI 3130xl Genetic Analyzer (Applied Biosystems). Segregation of variants in a family was assessed by sequencing of parental DNA samples.

## 3. Results

Clinical features present in the ALGS patients in our case series are summarized in Table 1. Liver biopsy of the proband number four with characteristic absence of intrahepatic bile ducts is shown in Figure 1A. A picture of proband number two with the typical facial dysmorphisms is presented in Figure 2. Identified pathogenic variants in the *JAG1* gene are summarized and characterized in Table 2. The patients were not related. All four mutation carriers are single heterozygotes. Two patients carry the as yet undefined pathological sequence variant in the *JAG1* gene.

The origin of the mutation in the first proband is not known, because the parents did not allow to be examined. In the second proband, the mutation was inherited from the mother. This pathogenic variant was described in 2019 by Gilbert et al. [19]. Until the time the child was diagnosed, the mother was kept under observation for adult gastroenterology because of hepatitis that was not accounted for. She suffered from aortic insufficiency, her facial features were typical of ALGS, she did not have embryotoxon posterior nor butterfly vertebra. The mutation of the third proband was inherited from the father who showed craniofacial dysmorphia typical of ALGS and in childhood had been under cardiologic observation for heart murmur. In adulthood, the patient’s cardiologic finding is normal. He had no other clinical symptoms of ALGS. The fourth proband had the above-described mutation occurring de novo [20]. None of the probands had neurovascular disorders and skin xanthomas. None had undergone transplantation of the liver or heart surgery. At present, the condition of all patients is good. 

## 4. Discussion

In the present study, we described the phenotype and genotype of four child ALGS1 probands. In one case, it was the above-mentioned nonsense mutation which resulted de novo [20]. In two cases, we discovered new frameshift mutations in the *JAG1* gene. Based on literary sources, about 30–50% of the affected individuals carry the inherited pathological sequence variant and about 50–70% carry the de novo variant [14,19]. 

Our probands suffer from predominant peripheral pulmonary stenosis in accordance with scientific literature [13,14]. Only the mother of one proband is being kept under observation for aortic insufficiency. Other ALGS-associated congenital heart defects, i.e., tetralogy of Fallot, atrial, or ventricular septal defect, stenosis, or coarctation of the aorta are not so frequent [14].

Posterior embryotoxon was found to be present in one of our probands. It is the most frequent type of eye defect in ALGS patients, often in association with 20p12 microdeletion. However, posterior embryotoxon is also present in 15% of the healthy population. Another less frequent eye affliction that ALGS patients suffer from is an anomaly of the pupils (eccentric or ectopic pupils), anomalous optic disc, chorioretinal atrophy, retinal pigment clumping, and the like [14].

One of our probands has a butterfly vertebra and one suffers from anomaly of the 12th rib. ALGS patients were found to have spinal fusion, decreased intervertebral spaces, spina bifida, absence of the 12th rib, and rarely craniosynostosis and radioulnar synostosis [14]. It is maintained that X-ray examination of the spine should be an integral part of the diagnostic panel of ALGS probands.

Some of the ALGS patients have kidney defects; probably more frequent in patients with pathological sequence variants in the *NOTCH2* gene. However, Kamath et al. did not find such a correlation in the eight patients that they analyzed [21]. In the present study, we described the ren arcuatus and kidney cysts. In the past, other structural anomalies were described and also renal tubular acidosis and stenosis of the renal artery [14].

Some rarer afflictions were also monitored in our patients: hypothyreosis, learning disorders, short stature, tendency for recurrent infections (in our case, pneumonia and inflammation of the middle ear) [14]. One proband showed a tendency for aggressive behaviour. 

It has been generally accepted that there is no correlation between the proband’s genotype and phenotype [22]. Some ALGS1 patients carry the mutation in the *JAG1* gene and the diagnosis could not be confirmed on the basis of diagnostic criteria only. Relatives of ALGS patients may show only individual symptoms of the disease, which implies a mild form of the disorder [15,23,24]. In our case, the father of ALGS1 proband no. 3 had only two symptoms: typical facial appearance and heart murmur in childhood. However, we failed to find out if the patient had a functional heart murmur or a minor heart defect in childhood. In 2001, Eldadah reported a family where only heart defects occurred and no liver disorders were described [25].

Molecular-genetic diagnostic confirmation of ALGS is of major importance for the family of the affected child. Clinical symptoms of ALGS are highly variable both within the same family and among different patients. The high detection rate of mutations in subjects with incomplete ALGS suggests the possibility that a substantial number of patients carrying a *JAG1* mutation are not clinically diagnosed with ALGS [23,24]. Examination is crucial for genetic consultancy and for future prenatal and pre-implantation diagnosis. Treatment of these patients is multidisciplinary and includes a paediatrician, hepatologist, cardiologist, ophthalmologist, nephrologist, nutrition therapist, roentgenologist, genetic counseling, and in some cases, a transplantation team. 

## 5. Conclusions

Appropriate counselling for ALGS is complicated due to the absence of clear genotype-phenotype correlations and marked variable expressivity, even when the variant is inherited (30–50% of cases). Therefore, great care must be taken in providing genetic counselling for ALGS, especially in the prenatal life.

## Figures and Tables

**Table 1:** Clinical features present in carriers of *JAG1* pathogenic variants.

Patient	Diagnosis	Peculiar	Cholestasis	Liver Biopsy	Heart Disease	Ocular	Skeletal	Renal	Others
	Age	Face				Anomalies	Anomalies	Anomalies	
1	16 month	yes	yes	intrahepatic bile duct	peripheral pulmonary	no	butterfly	no	learning disability
				paucity	artery stenosis		vertebrae		
2	6 years	yes	yes	intrahepatic bile duct	peripheral pulmonary	no	no	no	
				paucity	artery stenosis				
3	7 month	yes	yes	intrahepatic bile duct	peripheral pulmonary	no	no	ren	behavioral disorders
				paucity	artery stenosis			arcuatus	
4	3 month	yes	yes	intrahepatic bile duct	peripheral pulmonary	embryotoxon	rib	cystic	hypothyroidism
				paucity	artery stenosis	posterior	anomalies	disease	growth retardation

**Table 2:** Pathogenetic variants in *JAG1* found in patients with Alagille syndrome.

Patient	Identified Sequence Variants	Mutation	Exon	cDNA	Protein	Mutation	Reference
		Origin				Type	
1	gene *JAG1* (NM_000214.2):c.3189dupG in heterozygous state	not investigated	25	c.3189dupG	p.Asn1064Glufs*45	frameshift	this study
	novel, duplication						
2	gene *JAG1*(NM_000214.2): c.2039delG in heterozygous state	mother	16	c.2039delG	p.Gly680Alafs*63	frameshift	Gilbert et al., 2019 [19]
	deletion						
3	gene *JAG1* (NM_000214.2):c.1913delG in heterozygous state	father	15	c.1913delG	p.Cys638Leufs*105	frameshift	this study
	novel, deletion						
4	gene *JAG1* (NM_000214.2):c.2230C>T p.(Arg744Ter) in heterozygous state	de novo	18	c.2230C>T	p.Arg744Ter	nonsense	Krantz et al, 1998 [3]
	substitution						

The c. nomenclature is based on the cDNA sequence NM_000214.2.

## Abbreviations

ALGS	Alagille syndrome
ALGS1	Alagille syndrome type 1
ALGS2	Alagille syndrome type 2
